# Lean thinking to improve emergency department throughput at AORN Cardarelli hospital

**DOI:** 10.1186/s12913-018-3654-0

**Published:** 2018-12-03

**Authors:** Giovanni Improta, Maria Romano, Maria Vincenza Di Cicco, Anna Ferraro, Anna Borrelli, Ciro Verdoliva, Maria Triassi, Mario Cesarelli

**Affiliations:** 1 Department of Public Health, School of Medicine and Surgery, University of Naples ‘Federico II’, Naples, Italy; 2 Department of Medical and Surgical Sciences, University ‘Magna Graecia’ of Catanzaro, Catanzaro, Italy; 3 Responsible for the Programming and Health Planning Unit, Hospital “Antonio Cardarelli”, Naples, Italy; 4 Hospital “Antonio Cardarelli”, Naples, Italy; 5 Department of Electrical Engineering and Information Technology (DIETI), University of Naples ‘Federico II’, Naples, Italy; 6 Scientific Clinical Institutes Maugeri IRCCS, Telese, Italy

**Keywords:** Lean thinking, Emergency department, Public health, Quality improvement

## Abstract

**Background:**

Throughout the world, emergency departments (ED) are characterized by overcrowding and excessive waiting times. Furthermore, the related delays significantly increase patient mortality and make inefficient use of resources to the detriment of the satisfaction of employees and patients. In this work, lean thinking is applied to the ED of Cardarelli Hospital of Naples with the aim of increasing patient flow, improving the processes that contribute to facilitating the flow of patients through the various stages of medical treatment and eliminating all bottlenecks (queue) as well as all activities that generate waste.

**Methods:**

This project was performed at National Hospital A.O.R.N. A. Cardarelli of Naples. The historical times of access to the ED were analysed from January 2015 to June 2015, for a total of 16,563 records. Subsequently, starting in November 2015, corrective actions were implemented according to the Lean Approach. Data collected after the introduced improvements were collected from April 2016 to June 2016 and compared to those collected during the starting period.

**Results:**

The results acquired before application of the Lean Thinking strategy illustrated the as-is process with its drawbacks. An analysis of the non-added value activities was performed to identify the procedures that need to be improved. After implementation of the corrective actions, we observed a positive increase in the performance of the ED, quantified as percentages of hospitalized patients according to triage codes and waiting times.

**Conclusion:**

This work demonstrates the applicability of Lean Thinking to ED processes and its effectiveness in terms of increasing the efficiency of services and reducing waste (waiting times).

## Background

Recently, the National Health Service (NHS) sustainability challenge has been at the heart of political and technical debate. The challenge asks healthcare companies to seek management solutions to support technical and administrative processes with the aim of improving efficiency, productivity, and the appropriateness and quality of performance. Given this necessity, logical and managerial tools are applied to the management of healthcare companies, including private and public hospitals.

Today, the Lean Management Model is one of the most used models in industrial areas and rapidly spreading throughout the healthcare industry in the United States and Great Britain [1]. Lean Management originates from the Toyota Production System (TPS) [2] and aims to improve efficiency by eliminating particular types of waste (called muda, in Japanese) and activities, which absorb time and resources without adding any value to the production process [3].

The Lean Method exploits a set of tools to streamline processes and to eliminate unnecessary time-consuming steps. Lean thinking [4] seeks to enhance performance and meet customer needs by reducing complexity, improving process flow and removing unnecessary or non-value-added activities [5, 6], as has been well described by references [6–8].

The essential aspects of Lean systems are as follows: specified tasks, streamlined communication, simple process architecture, and hypothesis-driven problem solving [8, 9].

Since the 1970s, in healthcare competitions, factors such as zero defects, process time reduction, price and relevant customisation have gained great importance [10, 11].

To implement the Lean methodology, a healthcare organisation typically has to follow a theoretical path whose main stages are as follows [10]:

- Training Lean specialists and raising awareness about waste inside the processes.
- Determining the sequence of activities within the processes using tools such as Value Stream Map (VSM).
- Eliminating activities that do not add value to the process and designing the future state of the process.
- Improving the process (start over) through reactive teams that remove waste when it is identified.
- Using standardised Lean tools.

Lean principles have been successfully adapted to the healthcare environment, enabling hospitals and clinics to streamline their operations and to focus on value as perceived by their patients. Many healthcare organizations have sought the support of Lean manufacturing to improve their efficiency [12, 13].

Literature about Lean theory applied to healthcare is relatively recent. However, there are many scientific papers that deal with the subject [11] and demonstrate the benefits of applying Lean methodology in health systems [1, 14]. In particular, literature reviews indicate that Lean process tools have been successfully applied in the healthcare environment to a wide range of clinical situations, such as the choice of a prosthetic substitute, risk management and reduction of length of hospital stay [12, 15, 16].

Van Lent et at. [15] applied a Lean thinking approach for a hospital-based chemotherapy day unit. The method improved the process design and led to increased efficiency and more timely delivery of care. Bisgaard et al. [16] investigated the reduction of the length of stay of patients with chronic obstructive pulmonary disease. Their results illustrate the possibilities of improving quality while simultaneously reducing costs.

Other studies focused on improving the performance of the provided services [17, 18], developing and using quality indicators [19], reducing medical errors [20] and improving quality and efficiency of healthcare processes using both Lean, Six Sigma and further management or mathematical tools [21–30].

Lean tools have significant applicability in the Emergency Department (ED); throughout the world, EDs are challenged with problems of overcrowding and excessive waiting time [31–35].

Overcrowding and related delays increase patient mortality, decrease the satisfaction of employees and patient sand make inefficient the use of resources [1]. In addition, the ED is considered the heart of a hospital; thus, it critically impacts the organization.

Dickson et al. [17] used Lean manufacturing techniques in the ED in an effort to enhance patient and staff satisfaction. The implementation followed a six-step process of Lean education, ED observation, patient flow analysis, process redesign, and new process testing. Their results demonstrated a slight decrease in length of stay and a significant increase in patient satisfaction without raising the cost per patient. Furthermore, a recent review by Holden [31] on Lean applications indicates that Lean can help to decrease waiting times, length of stay, and the proportion of patients who leave the emergency room before a doctor has examined them.

The key elements for the success of Lean methodology in an ED is the ability to change, the involvement of the leadership, a clear mapping of processes and the introduction of small improvements that are long-term sustainable [17, 35, 36]. Such initiatives need to be realistic and improve patient satisfaction without overloading the work of staff.

The project here was performed at Hospital National Company “AORN Cardarelli” of Naples. Our project primarily aimed to improve the management of patients in the ED, in particular focusing on the following:

- Increase the flow to the client/patient, where the value is effectively measured by the time spent by the patient prior to receiving care within the ED;
- Improve the processes that contribute to the value of the clinical services and to facilitate transitions (flow) of patients through the various stages of medical treatment;
- Eliminate all bottlenecks, as well as all activities that generate waste and create queues

## Methods

The authors, in collaboration with a team of doctors and nurses, conducted a Lean Methodology assessment and thus conducted a study on lead times of all patients who had access to the Emergency Department of AORN Cardarelli before and after the application of Lean. The AORN A. Cardarelli is a hospital of national relief; it represents the largest hospital within South Italy and increasingly within the entire Italian peninsula. Indeed, its departments host thousands of patients every day. AORN A. Cardarelli plays a leading role in emergency healthcare, as it is a second-level Emergency-Acceptance Department, providing first aid services and emergency cares in many specialties. Furthermore, the Greater Burns Center, the Anti-Poison Center and the Center for Hepatic Transplants, (Regional Emergency Centers) are present. The Emergency Room (ER) of AORN Cardarelli is a part of a level II ED that manages 94,000 patients per year.

To study the constant overcrowding issue of the ED and the excessive patient waiting times, which greatly increase patient mortality and make inefficient use of resources at the expense of the employee and patient satisfaction, the Lean approach was applied. In particular, for this study, the historical times of accesses to the ER at AORN Cardarelli of Naples from January 2015 to June 2015 were collected, for a total of 16,563 records. Subsequently, the records were analysed and, starting from November 2015, some corrective actions (described in the following) were implemented. Finally, data post-Lean were collected in the period from April 2016 to June 2016.

The first phase of the study was a qualitative analysis, performed by means of several meetings with doctors and interviews with health care staff employed in the ER. In particular, five meetings took place; they involved the managing director, two nurses, a head nurse and a physician for each specialty.

In those meetings, the information, necessary to better understand the process was collected and subsequently analysed by quantitative indicators.

The result of this stage was knowledge of the entire process of admission and treatment of the patients in ER, briefly described in the following.

Upon the arrival of the patient at the ER, the patient is identified, and a triage colour code (red, green, yellow or white) is assigned to him/her after he or she reports his or her symptoms. Then, all patients undergo a reassessment. This revaluation takes place in another room assigned to the second triage after examining the vital signs. With the assignment of the colour code, the patient is assigned to one of the medical specialties (e.g., surgery, medical, orthopaedic, etc.). After the medical examination, instrumental diagnostic tests are activated and executed in the ED Radiology Department (adjacent to the ER) and/or in the Analysis Laboratory (outside the ED). Radiological results are brought by a nurse to the doctor’s desk and also sent by the computer system, whereas the laboratory reports are transmitted only by the informatics system. Then, if any specialist advice is required, it is requested. After tests and counselling have been completed, the patient is reassessed by the ER physician to determine discharge, hospitalization or transfer to a short observation room (in which they can remain 24–48 h).

At the end of the analysis of the ER processes, the preliminary stage of Lean assessment, a value stream mapping (VSM) was drafted. VSM is a key tool for identifying opportunities to reduce waste and more tightly integrate process steps, thus improving processing efficiency [4]. In a VSM, key people, resources, activities and information flows, required to deliver a product or service, are made explicit and depicted graphically.

**Fig. 1:**
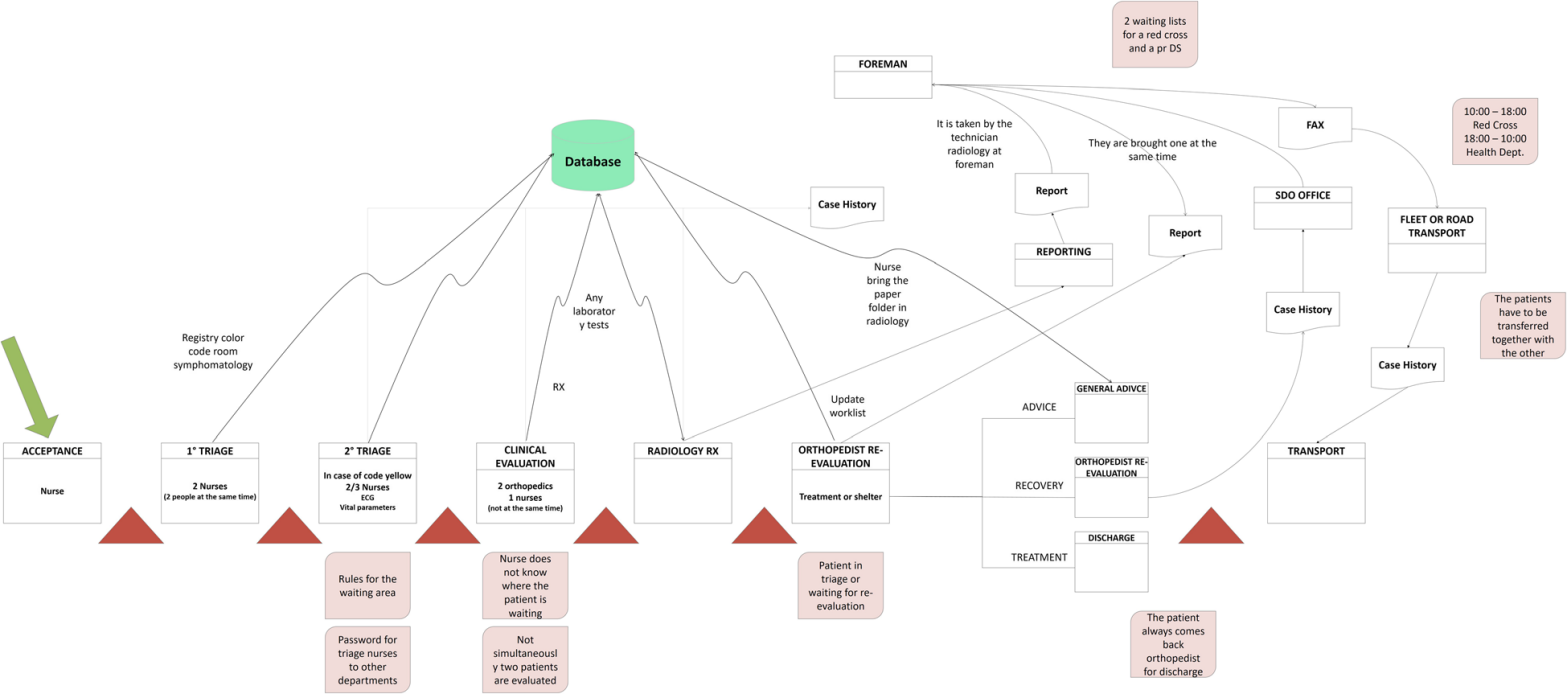
Value Stream Map

In our project, thanks to the cooperation of all the involved staff, the sequence of the workflow and the time required for each procedure were taken into consideration; the VSM shown in Fig. 1 was obtained.

This qualitative analysis was useful to detect the bottlenecks of the workflow and hence to choose the Lean principles on which to base future operations. The criticalities highlighted in this phase were the most time-consuming tasks, such as diagnostic examinations, triage code assignment, and counselling. For the sake of simplicity, the tasks were grouped into macro-categories: Path, Materials, Space, Communication, Logistic and Staff, as shown in Fig. 1.

Subsequently, the study went on with a more quantitative analysis. Literature reports [15, 24, 26] suggest that ED performance indicators should measure quality of triage, access times, and the quality of the service provided. Therefore, as performance measurements, we chose parameters primarily related to waiting times and service delivery depending on the colour code:

- **Percentage of patients with a yellow code examined within 30 min:** provides an estimation of the efficiency in managing yellow codes.
- **Percentage of patients with green code examined within 1 h:** provides an estimation of the efficiency in managing green codes.
- **Percentage of patients with green code not hospitalized with stay times ≤ 4 h**.
- **Percentage of patients sent to hospitalization with stay times ≤ 8 h**.
- **Lead time for colour codes:** The time from the patient’s arrival at the ER until the patient leaves the ER.

For the quantitative analysis, necessary data were extracted from the health information system commonly used by AORN Cardarelli. The obtained values (presented in the Results section) indicate that 99.8% of the patients were hospitalized within 8 h. Among green code cases, 94.5% are hospitalized in the first 4 h of stay.

At the end of the analysis of the current situation, waste was identified through direct observation of the Lean team on the field. As a result, a balance chart of the “as-is” process was developed. The process determined that the organization of the ER, and hence the workflow to which the patient is subjected, comprises five phases: triage, examination, diagnostic test, advice, and dismissal.

Each phase has added value and is also necessary; moreover, the balance chart technique highlighted the percentage of non-added value for each action. The non-added value was primarily due to delays in information delivery, which must be reduced to optimize the whole process.

What has emerged is that for triage, only 7 min were truly useful; the remaining time represented waiting for the successive phase and is the non-added value of the activity.

The examination took 13 min on average. Approximately, approximately 5 min were needed for the request of diagnostic tests and anamnesis; both activities are necessary, even if without value for the patient. In the remaining time, the patient waited for the diagnostic test (activity without added value and non-necessary).

The time required for the diagnostic test (radiography, echography, etc.) included approximately 12 min for the test itself, 10 min for the clinical report (necessary but non-added value activity) and more than 1 h elapsed in queues and delays before the report was available in the informatics system of the hospital.

The majority of the advice activity was due to waiting time; only 10 min were dedicated to the specialist medical examination and 10 to writing the report.

The last activity, dismissal, was the shortest, including approximately 8 min for the discharge procedure, 3 min on average for the exit report and approximately 7 min of waiting.

The activities involved in each phase of the process (patient stay in ER) are summarized in Table 1.

**Table 1:** Added- and non-added-value activities

Phase	Activity	Added value (min)	Non added value (min)	Non added value but necessary (min)
Triage	Triage	7		
	Waiting for examination		32	
Examination	Examination	13		
	Request for diagnostic tests			44
	Waiting for diagnostic tests		5	
Diagnostic Test	Diagnostic tests	12		
	Clinical report			60
	Clinical report available in the IT system		10	
Advice	Waiting for specialist advice		10	
	Specialist medical examination	10		
	Report writing			20
Dimissal	Waiting for the dismissal		3	
	Discharge procedure	8		
	Exit reporting			6

At the end of the analysis (values presented in the Results section), the average time elapsed for the whole process was 4 h and 18 min, of which 80% were due to non-value added activities, some of which were necessary but the majority of which were primarily waiting times.

Based on the above-mentioned analysis, conducted in accordance with Lean methodology, as introduced at the beginning of this section, in the second half of 2015, corrective actions were planned and implemented to simplify and speed up the procedures; hence, the process was redesigned to minimize or completely eliminate waste.

For example, a re-engineering process was performed to achieve the following: reduce the waiting time associated with report consultation; better organize the nurse stations with particular attention to drug layout to facilitate daily operations; and organize medical shifts to optimize time and resources.

As a result of the analysis of the “as-is” process, the corrective actions were primarily devoted to:

- standardize the work and reduce ambiguity;
- connect people who are interdependent;
- create seamless uninterrupted workflow through the process;
- allow staff to investigate process problems and to develop, test and implement countermeasures with a “scientific method” [9].

In particular, the key interventions reported in Table 2 were put in place. The main improvement actions concern the computerization of all the main procedures and the reorganization of the medical staff with well-defined roles and goals.

**Table 2:** Key Interventions

Type	Description	Time
5S Tecnique	Re-organization of the Nurses station	February 2016
Visual	Identification of the staff	November 2015
	Vertical and horizontal information plaques for shared spaces	December 2015
	Define the card for the patient waiting for first examination	March 2016
	Definition of the care path manager panel	March 2016
	Team leader becomes care path manager - goal setting	January 2016
Triage	Project presentation meeting	December 2015
Flow	Definition of the medical tutor role	December 2015
	Strategic management of medical shifts	January 2016
	Definition of a minimum number of medical examinations per shift for each location + periodic calculation	January 2016
	Sharing with staff and periodic reports	January 2016
Support	Define who needs the stretcher at the entrance, and limiting its use and replacing it when possible with a wheelchair	December 2015
Computerization	Create a web application that notifies the arrival of reports in real time	December 2015

With respect to the “computerization” intervention in Table 2, it is worth mentioning that the informatics system present at the AORN Cardarelli hospital before the Lean interventions consisted of the use of a first aid management system perfectly interfaced with the diagnostic services applications, including radiology, clinical analysis laboratory, and transfusion. Upon patient admission, the 1st triage code assignment is performed, and the personal data sheet is filled out on the ED management system. Depending on the assigned code, the patient is placed into different assistance and treatment paths. During the medical examination, the doctor writes the diagnosis and prescribes any diagnostic test, advice and possible therapy. Diagnostic examinations and consultations are requested through an informatics system called “Order Entry,” which maintains a communication between the ED management system and other applications. Once the examinations have been reported, they are automatically re-sent through the “Order Entry” to the ED management system, which stores the data until the doctor visualizes them. Possible advice is automatically sent in the same way. The therapy administered is recorded in the appropriate electronic record related to the patient, and the system proceeds with discharge or transfer of the patient by saving in the appropriate electronic record all of the necessary data and information for the discharge report.

The Takt Time [37] was then calculated for all of the activities to estimate the degree of satisfaction with the input demand. The Takt Time is an estimate of the desirable duration (from the patient’s point of view) of each activity of the healthcare process. The duration should be sufficient to satisfy the patient’s demand without creating queues or dead times (non-added-value activities). In essence, the Takt Time proves to be an interesting indicator of the patient’s satisfaction as well as of the efficiency of the process and makes it possible to define the rhythm through which the activities must be performed to meet the services’ demand.

Finally, the analysis was repeated after the collection of data concerning the period here called “post-Lean,” and the obtained results were compared. In particular, a statistical analysis by means of a *t*-test was performed on the identified performance indicators of the process.

## Results

In this section, the results obtained after the Lean methodology employment are presented.

Table 3 displays performance measurements before and after the Lean interventions.

**Table 3:** Performance Measurements (before and after Lean interventions)

	Before lean	After lean
Performance Measurements	Percentage (%)	Percentage (%)
Patients with a yellow code examined within 30 min	53,6	56,9
Patients with a green code examined within 1 h	52,6	54,3
Patients with a green code sent to hospitalization with a stay time ≤ 4 h	94,8	96,8
Patients sent to hospitalization with a stay time ≤ 8 h	99,8	99,8
Performance Measurements	Average ± std. (min)	Average ± std. (min)
Lead Time for red code	72 ± 36	71 ± 30
Lead Time for yellow code	151 ± 100	147 ± 67
Lead Time for green code	164 ± 116	163 ± 120
Lead Time for white code	160 ± 173	158 ± 156

**Fig. 2:**
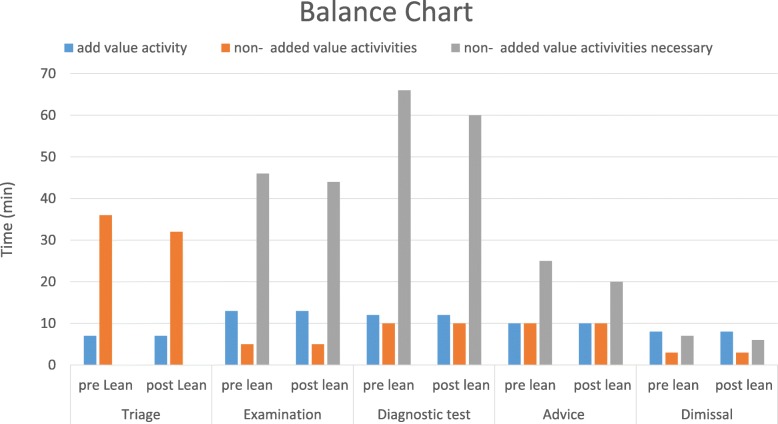
Balance chart before and after Lean interventions

In Fig. 2, a balance chart of the process before and after the Lean interventions is reported.

Based on Fig. 2, it is possible to observe that a reduction of the overall times (total of the five phases, triage, examination, diagnostic test, advice, and dismissal) was achieved through the implementation of the corrective actions after the Lean interventions. In particular, interventions devoted to improving the information system and the staff formation and collaboration lead to a reduction in delays in information delivery, identified as non-added-value activities.

At this point, it is worth remembering that the analysis of the “as-is” process highlighted that most of non-added value actions are waiting times, so we detailed these times for each colour code:

- Waiting time triage I - triage II,
- Waiting time triage I - taken into care,
- Waiting time taken into care - dismissal,
- Waiting time triage I - dismissal.

**Fig. 3:**
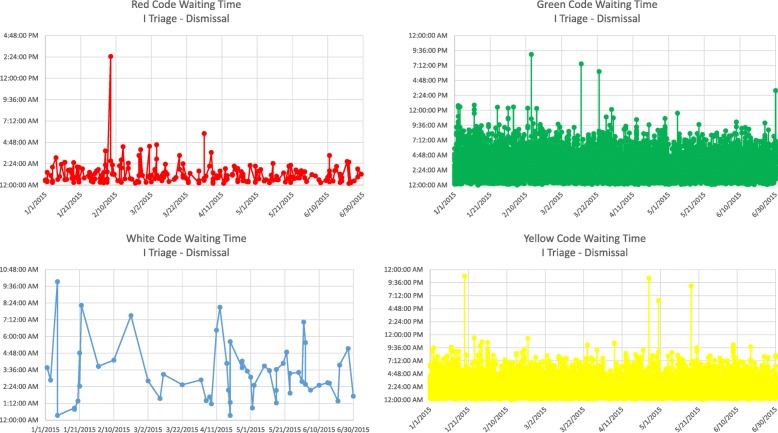
Control charts by colour code

Figure 3 depicts the stay time control chart of patients in the ER divided by colour code.

Finally, a comparison of the process before and after the application of the Lean methodology and the results of the *t*-test are reported in Table 4. The results confirm that the key interventions have significantly improved patient’s throughput times.

**Table 4:** Patient throughput times (before and after Lean interventions)

Variable	Average – STD pre-Lean (*N* = 16.563)	Average –STD post-Lean (*N* = 17.147)	*p*-value
Waiting time I triage – II triage	00:22:54–00:28:25	00:21:24–00:26:55	< 0.001
Waiting time I triage – taken into care	01:47:55–01:14:44	01:41:55–01:14:44	< 0.001
Waiting time taken into care – dismissal	02:31:02–01:27:18	02:19:12–01:03:20	< 0.001
Waiting time I triage – dismissal	04:18:57–01:52:11	04:01:07–01:02:57	< 0.001

## Discussion

Over time, the Lean approach, referring to the model adopted by Toyota exclusively for production, has expanded to involve the management and administrative processes of different companies: a true Lean Thinking encoded in Lean Management techniques. Today, Lean is not merely a set of tools but is based on individual involvement to create a culture of no ambiguity and quickly resolving problems. Lean consists of a management strategy applicable to all organizations because it involves the improvement of the processes. Lean is embodied in the identification of processes, in mapping the activities and in the continuous search and elimination of waste to produce more value with less consumption. In particular, to implement Lean thinking in a healthcare organisation, a theoretical path has to be followed whose main steps are as follows: training of staff and raising awareness about wastes involved in the processes; determining the precise sequence of activities, eliminating those without add value; improving the processes through agile and quick responses to remove waste as soon it is identified [11].

Many authors have analysed and proposed Lean applications in health care through case studies [11, 25–28]. In this work, we proposed a new, interesting case study based on the implementation of the Lean methodology to reduce waiting times in the ER and improve the flow of patients between the emergency room and recovery areas. We followed the theoretical path described mentioned, and, among principles and tools of the Lean methodology, we chose those considered the most important in healthcare management, such as VSM and 5S.

The aim was to govern the flows that the ED dictates to the entire operation of the Cardarelli hospital and to try to prevent useless waits in conditions that are often not compliant with care and assistance needs.

On the basis of the principles of Lean thinking, the medical directorate of the Cardarelli Hospital in Naples decided to review the patient’s path from the arrival in the ER to admission, recovery and discharge. This path is transversal to several operating units and includes processes pertinent to hospitals and others of territorial relevance. In detail, a re-engineering process that improves the management of medical reports and the creation of a web application that provides notifications on the arrival of reports in real time have been employed and/or developed to reduce the waiting time related to the doctors’ consultation. A series of operations, such as the definition of the role of the physicians and of a minimum number of visits per shift, as well as the installation of information plaques for shared spaces, has considerably reduced patient’s time from phase to phase. The non-wasted time is dedicated to the patient and provides the same service but at a higher level of quality and security. Moreover, delivering the same service in a shorter time ensures less waiting time not only for the patient involved but also for those others waiting.

The described theoretical path can be followed independently of the specific environment, which only influences the concrete and practice actions that must be implemented.

## Conclusions

In conclusion, the work demonstrated that by strictly following the theoretical path, choosing suitable tools, and applying the principles and methods of Lean Thinking to the healthcare processes, it is possible to increase the efficiency of services, reduce waste in terms of waiting times, and improve the quality of the working environment for operators.

## Abbreviations

ED: Emergency department
ER: Emergency room
NHS: National health service
TPS: Toyota production system
VSM: Value stream map
